# Improving indicator-condition guided testing for HIV in the hospital setting (PROTEST 2·0): A multicenter, interrupted time-series analysis

**DOI:** 10.1016/j.lanepe.2022.100515

**Published:** 2022-10-07

**Authors:** Saskia J. Bogers, Maarten F. Schim van der Loeff, Anders Boyd, Udi Davidovich, Marc van der Valk, Kees Brinkman, Kim Sigaloff, Judith Branger, Nejma Bokhizzou, Godelieve J. de Bree, Peter Reiss, Jan E.A.M. van Bergen, Suzanne E. Geerlings, HIV Transmission Elimination AMsterdam (H-TEAM) Initiative, T. van Benthem, D. Bons, G.J. de Bree, P. Brokx, U. Davidovich, F. Deug, S.E. Geerlings, M. Heidenrijk, E. Hoornenborg, M. Prins, P. Reiss, A. van Sighem, M. van der Valk, J. de Wit, W. Zuilhof, N. Schat, D. Smith, M. van Agtmael, J. Ananworanich, D. van de Beek, G.E.L. van den Berk, D. Bezemer, A. van Bijnen, J.P. Bil, W.L. Blok, S.J. Bogers, M. Bomers, A. Boyd, W. Brokking, D. Burger, K. Brinkman, N. Brinkman, M. de Bruin, S. Bruisten, L. Coyer, R. van Crevel, M. Dijkstra, Y.T. van Duijnhoven, A. van Eeden, L. Elsenburg, M.A.M. van den Elshout, E. Ersan, P.E.V. Felipa, T.B.H. Geijtenbeek, J. van Gool, A. Goorhuis, M. Groot, C.A. Hankins, A. Heijnen, M.M.J Hillebregt, M. Hommenga, J.W. Hovius, Y. Janssen, K. de Jong, V. Jongen, N.A. Kootstra, R.A. Koup, F.P. Kroon, T.J.W. van de Laar, F. Lauw, M.M. van Leeuwen, K. Lettinga, I. Linde, D.S.E. Loomans, I.M. van der Lubben, J.T. van der Meer, T. Mouhebati, B.J. Mulder, J. Mulder, F.J. Nellen, A. Nijsters, H. Nobel, E.L.M. Op de Coul, E. Peters, I.S. Peters, T. van der Poll, O. Ratmann, C. Rokx, M.F. Schim van der Loeff, W.E.M. Schouten, J. Schouten, J. Veenstra, A. Verbon, F. Verdult, J. de Vocht, H.J. de Vries, S. Vrouenraets, M. van Vugt, W.J. Wiersinga, F.W. Wit, L.R. Woittiez, S. Zaheri, P. Zantkuijl, A. Żakowicz, M.C. van Zelm, H.M.L. Zimmermann

**Affiliations:** a Amsterdam UMC location University of Amsterdam, Internal Medicine, Meibergdreef 9, Amsterdam, the Netherlands; b Amsterdam Institute for Infection and Immunity, Infectious Diseases, Amsterdam, the Netherlands; c Amsterdam Public Health Research Institute, Quality of Care, Amsterdam, the Netherlands; d Department of Infectious Diseases, Public Health Service of Amsterdam, Amsterdam, the Netherlands; e Stichting hiv monitoring, Amsterdam, the Netherlands; f Department of Internal Medicine, Onze Lieve Vrouwe Gasthuis, Amsterdam, the Netherlands; g Amsterdam UMC location Vrije Universiteit Amsterdam, Internal Medicine, De Boelelaan 1117, Amsterdam, the Netherlands; h Department of Internal Medicine, Flevoziekenhuis, Almere, the Netherlands; i Department of Internal Medicine, BovenIJ ziekenhuis, Amsterdam, the Netherlands; j Amsterdam institute for Global Health and Development, Amsterdam, the Netherlands; k Amsterdam UMC location University of Amsterdam, Global Health, Meibergdreef 9, Amsterdam, the Netherlands; l Amsterdam UMC location University of Amsterdam, General Practice, Meibergdreef 9, Amsterdam, the Netherlands; m STI AIDS Netherlands, Amsterdam, the Netherlands

**Keywords:** HIV, HIV testing, Indicator condition, Tuberculosis, Cervical carcinoma, Cervical dysplasia, Vulvar carcinoma, Vulvar dysplasia, Lymphoma, Hepatitis B, Hepatitis C, Neuropathy, Intervention, Medical education, Diagnostics

## Abstract

**Background:**

Indicator-condition (IC) guided HIV testing is a feasible and cost-effective strategy to identify undiagnosed people living with HIV (PLHIV), but remains insufficiently implemented. We aimed to promote IC-guided HIV testing in seven ICs.

**Methods:**

Relevant departments in five hospitals of the Amsterdam region participated. HIV testing among adult patients without known HIV infection but with an IC was assessed using electronic health records during pre-intervention (January 2015–June 2020) and intervention (July 2020–June 2021) periods. The multifaceted intervention included audit and feedback. The primary endpoint was HIV testing ≤3 months before or after IC diagnosis and the effect of the intervention was evaluated using segmented Poisson regression.

**Findings:**

Data from 7986 patients were included, of whom 6730 (84·3%) were diagnosed with an IC in the pre-intervention period and 1256 (15·7%) in the intervention period. The proportion HIV tested ≤3 months before or after IC diagnosis increased from 36.8% to 47.0% (adjusted risk ratio [RR]= 1.16, 95% CI=1.03–1.30, *p*=0.02). For individual ICs, we observed significant increases in HIV testing among patients with cervical cancer or intraepithelial neoplasia grade 3 (adjusted RR=3.62, 95% CI=1.93–6.79) and peripheral neuropathy (adjusted RR=2.27 95% CI=1.48–3.49), but not the other ICs. Eighteen of 3068 tested patients were HIV positive (0.6%).

**Interpretation:**

Overall IC-guided testing improved after the intervention, but not for all ICs. Variations in effect by IC may have been due to variations in implemented developments, but the effect of separate elements could not be assessed.

**Funding:**

HIV Transmission Elimination Amsterdam (H-TEAM) initiative, Aidsfonds (grant number: P-42702).

**Research in context**

**Evidence before this study**

Prevention of HIV transmission through HIV diagnosis and treatment is key to end the HIV epidemic. A feasible and cost-effective strategy is to test for HIV in patients with indicator conditions (ICs), that are associated with HIV. However, this strategy is still insufficiently implemented in Western countries. Patients with ICs may present themselves across both infectious disease and non-infectious disease specialties in the hospital setting, but knowledge among healthcare professionals (HCP) of this testing strategy varies by specialty. We searched Ovid MEDLINE and Embase from inception up until April 29th, 2022, using various terms for ‘indicator condition’, ‘HIV testing’ and ‘intervention’ or ‘educational’ or ‘improving’ to identify studies aiming to improve IC-guided testing for HIV in the hospital setting. No language restrictions were used. Reference lists of included references were additionally searched. We identified 115 references of which 4 full-text articles and 9 short reports/conference abstracts reported on the effect of implemented interventions. The mean increase in HIV testing among eligible patients was 23% after implementation (range -6% to 60%). Interventions that were most effective at increasing HIV testing were those that employed a combination of an educational intervention for HCP including audit and feedback as well as structural changes such as routine/opt-out testing, changes to order-sets or guideline adaptations. Conversely, isolated educational interventions or implementation of routine testing alone were least effective.

**Added value of this study**

Our multicenter intervention study confirmed that using a multifaceted intervention including an educational intervention with audit and feedback, as well as structural changes including guideline adaptations, electronic prompts, reflex testing and visual prompts effectively increased IC-guided HIV testing in the hospital setting. However, the effect may depend on variations in implementation by setting. We also confirmed this testing strategy's cost-effectiveness to identify undiagnosed people living with HIV in a high-income setting, as the HIV positivity percentage observed exceeded the cost- effectiveness threshold.

**Implications of all the available evidence**

Multifaceted interventions employing a combination of educational interventions and structural solutions to support HIV testing effectively increase IC-guided HIV testing, which is a cost-effective strategy to identify undiagnosed people living with HIV.

Alt-text: Unlabelled box

## Introduction

Timely HIV diagnosis is key to our efforts in ending the HIV epidemic. Earlier diagnosis is associated with numerous individual health benefits, such as decreased morbidity, hospital admissions, and mortality,^1^, ^2^, ^3^ while also preventing onward HIV transmission.^4^ One feasible and cost-effective strategy is to routinely test patients diagnosed with an HIV indicator condition (IC).^5^, ^6^, ^7^ ICs are AIDS-defining illnesses and HIV-associated conditions in which ≥1 per 1000 individuals (≥0.1%) have undiagnosed HIV. They include conditions that share the same transmission route as HIV and conditions commonly seen with HIV-associated immunosuppression.^8^ However, IC-guided HIV testing is still being insufficiently implemented in many Western countries a decade after its global introduction.^9^ Adopting systematic IC-guided testing, and creating awareness of this strategy among involved specialties is an important first step in improving its implementation.^9^

Overall, 24,000 people were estimated to be living with HIV in the Netherlands in 2020, of which an estimated 1640 (7%) remained undiagnosed. An estimated 6420 people living with HIV resided in Amsterdam, including 300 (5%) undiagnosed individuals. It is estimated that 90% of HIV transmissions in the Netherlands come from persons with undiagnosed HIV.^10^^,^^11^ More appropriate HIV testing strategies could therefore help to reach our goal of ending the HIV epidemic by 2030.^10^ We introduced a multifaceted intervention in five hospitals of the Amsterdam region to promote IC-guided HIV testing.^12^ Our objectives were to (1) generate awareness about ICs and the importance of IC-guided HIV testing amongst physicians working in hospitals, and (2) improve HIV testing in patients with ICs amongst different medical specialties in the hospital setting. In this study, we aimed to evaluate the effect of this intervention on HIV testing in patients diagnosed with ICs.

## Methods

### Study design and setting

We conducted a multicentre intervention study at two university hospitals, two non-academic teaching hospitals and one non-teaching hospital. The study protocol has been described elsewhere and registered with the Dutch Trial registry.^12^ Reporting was done in accordance with the Revised Standards for Quality Improvement Reporting Excellence (SQUIRE 2.0) guidelines (Supplementary table 1). During the pre-intervention phase, data on IC-guided HIV testing from January 2015 through June 2020 were collected. For all hospitals and departments, the intervention started on July 1, 2020. A repeat assessment of IC-guided HIV testing was performed in all settings from July 2020 through June 2021. We refer to this one-year period as the intervention period, which included the roll-out of the interventions as well as the assessment of its effects. An *a priori* selection of seven ICs was included based on their relatively high incidence and the fact that they are managed by several medical specialties (i.e. pulmonology, gynaecology, haematology, gastroenterology and neurology) and were expected to vary in the proportion of patients that were tested for HIV prior to the intervention. These ICs were: tuberculosis (TB), cervical cancer or cervical intraepithelial neoplasia grade III (CC/CIN-3), vulvar cancer or vulvar intraepithelial neoplasia grade III (VC/VIN-3), malignant lymphoma (ML), hepatitis B virus infection (HBV), hepatitis C virus infection (HCV) and peripheral neuropathy (PN).

### Intervention strategy

The intervention primarily consisted of a tailored educational intervention session using audit and feedback, taking place at each relevant specialty in each participating hospital during the intervention period. The sessions were scheduled by local physicians and conducted live or through video-conferencing based on the department's preferences and in accordance with any locally implemented COVID-19 measures. Attendants were medical specialists, residents and interns. To optimize the efficacy of the intervention, we employed a multifaceted strategy consisting of various elements (Table 1).^12^

**Table 1:** Elements of the multifaceted strategy to promote indicator condition-guided testing for HIV, Amsterdam region, the Netherlands, 2020.

**Pre-intervention: Assessment of barriers, facilitators and opportunities for improvement**
- Assessment of HIV testing recommendations in local and national IC specialty guidelines
- Dissemination of an online questionnaire among medical specialists and residents from relevant specialties (pulmonology, gynaecology, haematology, gastroenterology and neurology) to assess barriers and facilitators for IC-guided HIV testing
- Conduction of semi-structured interviews in a convenience sample of at least one physician per specialty to assess opportunities for improved HIV testing strategies
**Intervention: Educational sessions and further implementation elements**
- Reporting of competitive feedback on HIV testing behaviour per specialty, compared to other hospitals:
- Reporting results of proportion of patients tested for HIV in the pre-intervention period for the relevant IC per specialty
- Reporting results from pre-intervention phase: barriers, facilitators and opportunities for improvement
- Reporting evidence on IC-guided HIV testing and up-to-date information on HIV epidemiology, testing and treatment
- Interactive discussion on strategies to further improve HIV testing in the department, such as:
- Adding HIV testing recommendations to specialty guidelines
- Adding HIV testing to the standard laboratory order sets for outpatients
- Implementing electronic prompts for HIV testing
- Implementing reflex testing for HIV in the case of IC diagnosis
- Dissemination of materials (pocket cards and posters) on IC-guided HIV testing and specialty-specific information
- Assisting in implementation of any structural solutions for routine or improved HIV testing that were proposed during the intervention phase
- Dissemination of a newsletter including a summary of the information from the intervention phase, a discussion of the most commonly reported barriers for appropriate HIV testing, and a 3-minute educational video on HIV testing of patients with a relevant IC

IC: indicator condition.

### Patient eligibility

In each participating hospital, patients 18 years or over were identified using national disease billing codes. Patients without one of the selected definitive IC diagnoses, those with a known HIV infection prior to IC diagnosis, and those diagnosed and treated for their IC at another hospital were excluded by reviewing the patients’ electronic health records (EHR). However, patients who were referred for a second opinion or transferred for treatment after IC diagnosis were included. Several IC-specific inclusion criteria were used (Supplementary table 2). All eligible patients from university hospital 1 were included in the dataset. For all other hospitals, a random sample of 500 patients per IC was screened for eligibility if >500 patients were identified. This sampling was done to maintain a manageable workload as the added precision of more than 500 inclusions is negligible.^12^

### Data collection

Data on patient demographics, diagnosed IC, and HIV testing (if any) were extracted from the EHRs of eligible patients, which contain integrated hospital laboratory data, using a standardized data collection form (Supplementary table 3). For HIV testing, all laboratory records, scanned documents and patient notes were searched for evidence of any HIV test performed. If there was no evidence of HIV testing, reasons for not testing for HIV were sought and recorded if available. Female patients with a recorded pregnancy in the Netherlands after January 1, 2004 were assumed to have been tested for HIV by their midwife during antenatal care, as the number opting out of this universal screening method is negligible.^13^ EHR reviews and data processing were performed by several junior researchers and a random sample of ≥10% per IC was checked for agreement by the primary research physician (SJB). All data were processed using Castor (Castor Electronic Data Capture, Amsterdam, the Netherlands).

### Outcomes

The primary outcome was the proportion of patients diagnosed with an IC who were tested for HIV within 3 months before or after IC diagnosis. Secondary outcomes were the proportion of patients tested for HIV before initiating treatment for their IC, the proportion of patients not tested within 3 months before or after IC diagnosis where a reason for not testing was reported, the percentage testing HIV positive within 3 months before or after IC diagnosis, the proportion of new HIV diagnoses that were late stage infections (defined as CD4 count <350 cells/mm^3^ in this study), the proportion HIV tested within 6 months before or after IC diagnosis, and the proportion of patients diagnosed with an IC that were ever tested for HIV before or up to 6 months after IC diagnosis.

### Statistical analysis

Categorical data were summarised using frequencies and percentages, and continuous data as means and standard deviations (SD) or medians and interquartile ranges (IQR). Variable distributions were compared between patients diagnosed with an IC in the pre-intervention versus intervention phase using unpaired *t*-tests or Mann-Whitney U tests for continuous data and *X*^2^ or Fisher-exact tests for categorical data. A binomial probability test was performed to compare the observed percentage HIV positive in our study to the 0.1% positivity that has been identified as the cost-effectiveness threshold for routine HIV testing in previous studies. We modelled the overall proportion tested for HIV as a function of calendar time (in quarter-year periods) and intervention period (pre-intervention versus intervention) using a segmented (i.e., interrupted) time-series Poisson regression model. We evaluated the effect of the intervention from the intervention period term, which represents the log relative change in proportion tested from the intervention versus pre-intervention periods. The null hypothesis of no change in proportion was tested using a Wald χ^2^ test. We estimated this model for both the overall population, as well as each IC separately and each IC per hospital separately. An average number of 31 patients per IC per quarter-year was determined sufficient to reach >95% power to determine the anticipated 18–20% increase in HIV testing due to the intervention.^12^ Patient characteristics that were deemed potential confounders (i.e., age, sex, socio-economic status [SES] as derived from patient's 4-digit postal-code and stratified in low SES, intermediate SES and high SES based on national tertiles, and pregnant at IC diagnosis) were added to the regression model. The outcome was not over-dispersed (i.e. modeling the outcome with negative-binomial regression did not improve fit). Additionally, a separate analysis including a random intercept for hospital was performed to account for the variation between hospitals. We performed two sensitivity analyses: (1) the proportion HIV tested within 3 months after IC diagnosis only was used as the endpoint to evaluate the effect of the intervention on HIV testing as a reflex to diagnosing an IC, and (2) patients who died within 3 months after IC diagnosis were excluded to evaluate the potential effect of immortality bias. A *p*-value of <0.05 was considered statistically significant. All analyses were performed using Stata (v15.1, StataCorp, College Station, TX, USA).

### Role of the funding sources

The funders of the study had no role in study design, data collection, data analysis, data interpretation, or writing of the report. The corresponding author had full access to all data in the study and had final responsibility for the decision to submit for publication.

### Ethical considerations

All eligible patients were given the opportunity to opt-out of the use of their data. The Medical Ethics Committee of the Amsterdam University Medical Centers location University of Amsterdam determined that this study did not meet the definition of medical research involving human subjects under Dutch law.

## Results

### Study population

**Figure 1:**
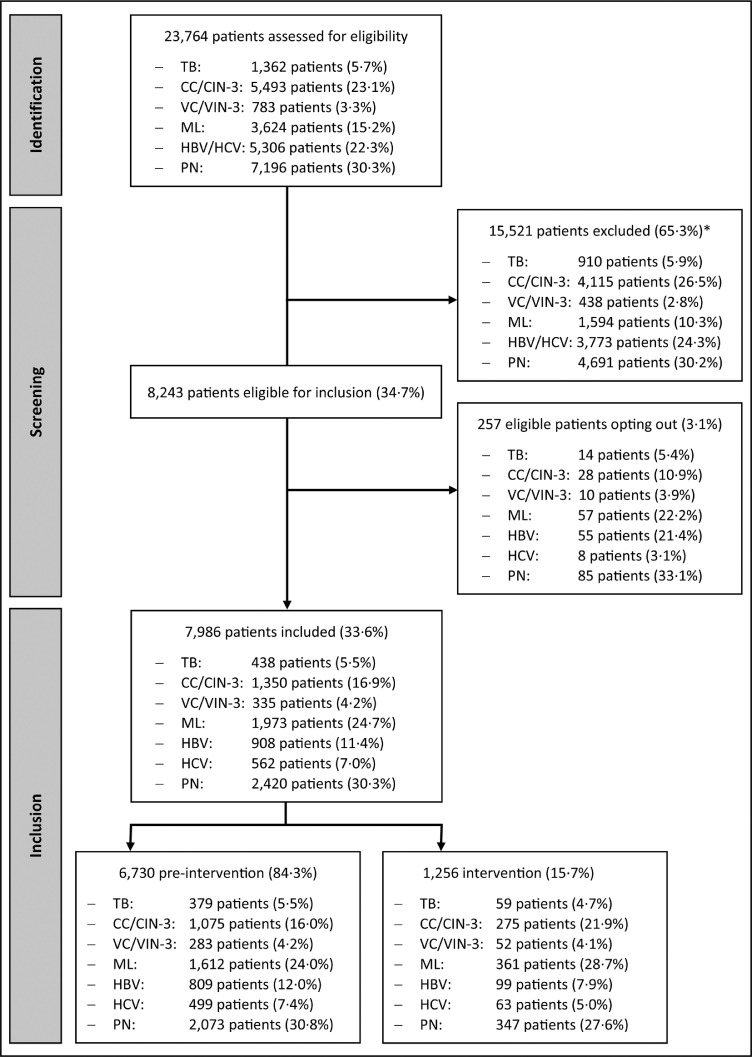
Flowchart of identification, screening and inclusion of data of patients diagnosed with indicator conditions in 5 hospitals in the region of Amsterdam, 2015–2021. *Reasons for exclusion were: no definitive indicator condition diagnosis (53.4%), indicator condition diagnosis outside study period (18.3%), indicator condition-specific exclusion criteria (18.0%), diagnosed and treated for the indicator condition at another hospital (6.6%), and known HIV infection prior to IC diagnosis (3.7%). HBV and HCV could not be reported separately in the identification and screening phase as they have a shared disease billing code. TB: tuberculosis, CC/CIN-3: cervical cancer or intraepithelial neoplasia grade III, VC/VIN-3: vulvar cancer or intraepithelial neoplasia grade III, ML: malignant lymphoma, HBV: hepatitis B virus infection, HCV: hepatitis C virus infection, PN: peripheral neuropathy.

The EHRs of 23,764 patients were assessed for eligibility and data of 7986 patients were included in the analysis, including 6730 patients (84.3%) in the pre-intervention period and 1256 (15.7%) in the intervention period (Figure 1). A mean of 44 patients per IC per quarter-year were included. More patients died within 3 months after IC diagnosis in the intervention period compared to the pre-intervention period, while other patient characteristics were comparable. However, additional differences in patient characteristics were observed when stratified by IC (Table 2).

**Table 2:** Characteristics of included patients diagnosed with indicator conditions in five hospitals in the region of Amsterdam before- and after intervention, overall and by indicator condition, 2015–2021.

	Overall	Before intervention	After intervention	*p* value
Overall	**(*n*=7986)**	**(*n*=6730)**	**(*n*=1256)**	
Sex				0.24
Female	4488 (56.2%)	3763 (55.9%)	725 (57.7%)	
Male	3498 (43.8%)	2967 (44.1%)	531 (42.3%)	
Pregnant at IC diagnosis^a^	150 (3.3%)	128 (3.4%)	22 (3.0%)	0.61
Age at IC diagnosis, y	56 (41–68)	56 (41–68)	58 (41–69)	0.18
Socio-economic status^b^				0.49
Low	2897 (36.6%)	2454 (36.8%)	443 (35.4%)	
Intermediate	1833 (23.2%)	1529 (22.9%)	304 (24.3%)	
High	3189 (40.3%)	2683 (40.3%)	506 (40.4%)	
Died ≤3 months after IC diagnosis	133 (1.7%)	103 (1.5%)	30 (2.4%)	0.03
Hospital of inclusion				<0.001
University hospital 1	3306 (41.4%)	2945 (43.8%)	361 (28.7%)	
University hospital 2	1083 (13.6%)	919 (13.7%)	164 (13.1%)	
Teaching hospital 1	1891 (23.7%)	1531 (22.8%)	360 (28.7%)	
Teaching hospital 2	786 (9.8%)	612 (9.1%)	174 (13.9%)	
Non-teaching hospital 1	920 (11.5%)	723 (10.7%)	197 (15.7%)	
Tuberculosis	**(*n*=438)**	**(*n*=379)**	**(*n*=59)**	
Sex				0.40
Female	164 (37.4%)	139 (36.7%)	25 (42.4%)	
Male	274 (62.6%)	240 (63.3%)	34 (57.6%)	
Pregnant at IC diagnosis^a^	3 (1.8%)	3 (2.2%)	0 (0%)	0.46
Age at IC diagnosis, y	42 (31–58)	42 (31–58)	45 (32–57)	0.57
Socio-economic status^b^				0.66
Low	228 (53.2%)	200 (53.9%)	28 (48.3%)	
Intermediate	80 (18.7%)	67 (18.1%)	13 (22.4%)	
High	121 (28.2%)	104 (28.0%)	17 (29.3%)	
Died ≤3 months after IC diagnosis	9 (2.1%)	9 (2.4%)	0 (0%)	0.23
Cervical cancer or CIN-3	**(*n*=1350)**	**(*n*=1075)**	**(*n*=275)**	
Pregnant at IC diagnosis^a^	41 (3.0%)	34 (3.2%)	7 (2.6%)	0.59
Age at IC diagnosis, y	41 (32–52)	40 (32–51)	41 (32–56)	0.23
Socio-economic status^b^				0.33
Low	468 (34.9%)	368 (34.5%)	100 (36.4%)	
Intermediate	345 (25.7%)	268 (25.1%)	77 (28.0%)	
High	530 (39.5%)	432 (40.5%)	98 (35.6%)	
Died ≤3 months after IC diagnosis	15 (1.1%)	8 (0.7%)	7 (2.6%)	0.01
Vulvar cancer or VIN-3	**(*n*=335)**	**(*n*=283)**	**(*n*=52)**	
Pregnant at IC diagnosis^a^	1 (0.3%)	1 (0.4%)	0 (0%)	0.67
Age at IC diagnosis, y	70 (59–79)	71 (59–80)	69 (59–76)	0.44
Socio-economic status^b^				0.48
Low	134 (40.1%)	117 (41.5%)	17 (32.7%)	
Intermediate	112 (33.5%)	93 (33.0%)	19 (36.5%)	
High	88 (26.4%)	72 (25.5%)	16 (30.8%)	
Died ≤3 months after IC diagnosis	6 (1.8%)	6 (2.1%)	0 (0%)	0.29
Malignant lymphoma	**(*n*=1973)**	**(*n*=1612)**	**(*n*=361)**	
Sex				0.71
Female	837 (42.4%)	687 (42.6%)	150 (41.6%)	
Male	1136 (57.6%)	925 (57.4%)	211 (58.5%)	
Pregnant at IC diagnosis^a^	10 (1.2%)	7 (1.0%)	3 (2.0%)	0.32
Age at IC diagnosis, y	61 (50–71)	61 (49–71)	62 (50–70)	0.89
Socio-economic status^b^				0.55
Low	598 (30.6%)	494 (31.0%)	104 (28.9%)	
Intermediate	477 (24.4%)	392 (24.6%)	85 (23.6%)	
High	877 (44.9%)	706 (44.4%)	171 (47.5%)	
Died ≤3 months after IC diagnosis	89 (4.5%)	70 (4.3%)	19 (5.3%)	0.45
Hepatitis B virus infection	**(*n*=908)**	**(*n*=809)**	**(*n*=99)**	
Sex				0.53
Female	377 (41.5%)	333 (41.2%)	44 (44.4%)	
Male	531 (58.5%)	476 (58.8%)	55 (55.6%)	
Pregnant at IC diagnosis^a^	81 (21.5%)	72 (21.6%)	9 (20.5%)	0.86
Age at IC diagnosis, y	41 (33–52)	41 (33–52)	40 (32–52)	0.97
Socio-economic status^b^				0.19
Low	479 (53.1%)	422 (52.5%)	57 (57.6%)	
Intermediate	163 (18.1%)	142 (17.7%)	21 (21.2%)	
High	261 (28.9%)	240 (29·9%)	21 (21.2%)	
Died ≤3 months after IC diagnosis	3 (0.3%)	2 (0.3%)	1 (1.0%)	0.21
Hepatitis C virus infection	**(*n*=562)**	**(*n*=499)**	**(*n*=63)**	
Sex				0.10
Female	186 (33.1%)	171 (34.3%)	15 (23.8%)	
Male	376 (66.9%)	328 (65.7%)	48 (76.2%)	
Pregnant at IC diagnosis^a^	7 (3.8%)	6 (3.5%)	1 (6.7%)	0.54
Age at IC diagnosis, y	53 (43–60)	53 (44–60)	50 (38–59)	0.09
Socio-economic status^b^				0.02
Low	223 (40.7%)	206 (42.5%)	17 (27.0%)	
Intermediate	121 (22.1%)	100 (20.6%)	21 (33.3%)	
High	204 (37.2%)	179 (36.9%)	25 (39.7%)	
Died ≤3 months after IC diagnosis	1 (0.2%)	1 (0.2%)	0 (0%)	0.72
Peripheral neuropathy	**(*n*=2420)**	**(*n*=2073)**	**(*n*=347)**	
Sex				0.11
Female	1239 (51.2%)	1075 (51.9%)	164 (47.3%)	
Male	1181 (48.8%)	998 (48.1%)	183 (52.7%)	
Pregnant at IC diagnosis^a^	7 (0.6%)	5 (0.5%)	2 (1.2%)	0.23
Age at IC diagnosis, y	64 (54–73)	64 (54–72)	66 (56–74)	0.01
Socio-economic status^b^				0.33
Low	767 (31.8%)	647 (31.4%)	120 (34.7%)	
Intermediate	535 (22.2%)	467 (22.6%)	68 (19.7%)	
High	1108 (46.0%)	950 (46.0%)	158 (45.7%)	
Died ≤3 months after IC diagnosis	10 (0.4%)	7 (0.3%)	3 (0.9%)	0.16

Data are depicted as n (%) or median (IQR).

a Percentages are calculated using the number of female patients as denominator.

b Overall, 67 patients had a missing socio-economic status value; 64 in the pre-intervention and 3 in the intervention period. CIN-3: Cervical intraepithelial neoplasia grade III. IC: Indicator condition. VIN-3: vulvar intraepithelial neoplasia grade III.

### Intervention

Overall, 26 educational intervention sessions were conducted among the five different specialties in the five participating hospitals, and a total of 384 physicians attended. Median number of attendees per session was 13 (IQR 8-20). Additional developments to improve IC-guided HIV testing occurred as a result of the educational intervention in several hospitals and specialties (Table 3).

**Table 3:** Additional developments that occurred as a result of the educational intervention to promote indicator condition-guided testing for HIV, Amsterdam region, the Netherlands, 2020–2022.

Development	Time of implementation
**All participating hospitals**	
Electronic prompts for HIV testing in electronic health records in the case of tuberculosis, hepatitis B virus infection and hepatitis C virus infection diagnoses	March 2021 for both university hospitals, March 2022 for both teaching hospitals and the non-teaching hospital^a^
**University hospital 1**	
Recommendation of HIV testing in local protocol for patients diagnosed with cervical carcinoma	December 2020
Reflex testing for HIV in the case of hepatitis B virus infection or hepatitis C virus infection	November 2021^a^
Addition of HIV testing as part of standard orders for newly diagnosed malignant lymphoma patients	September 2020
**University hospital 2**	
Addition of HIV testing as part of standard orders for newly diagnosed malignant lymphoma patients	September 2020
**Teaching hospital 1**	
Recommendation of HIV testing in the local protocol for patients diagnosed with cervical carcinoma	April 2021
Recommendation of HIV testing in the local protocol for patients diagnosed with peripheral neuropathy	January 2021
**Teaching hospital 2**	
Routine check of HIV testing before start of therapy in all malignant lymphoma patients by oncology nurse	December 2020

a Implementation occurred after the intervention's effect assessment was concluded, and is therefore not reflected in our findings.

### Proportion HIV tested within 3 months before or after IC diagnosis

**Figure 2:**
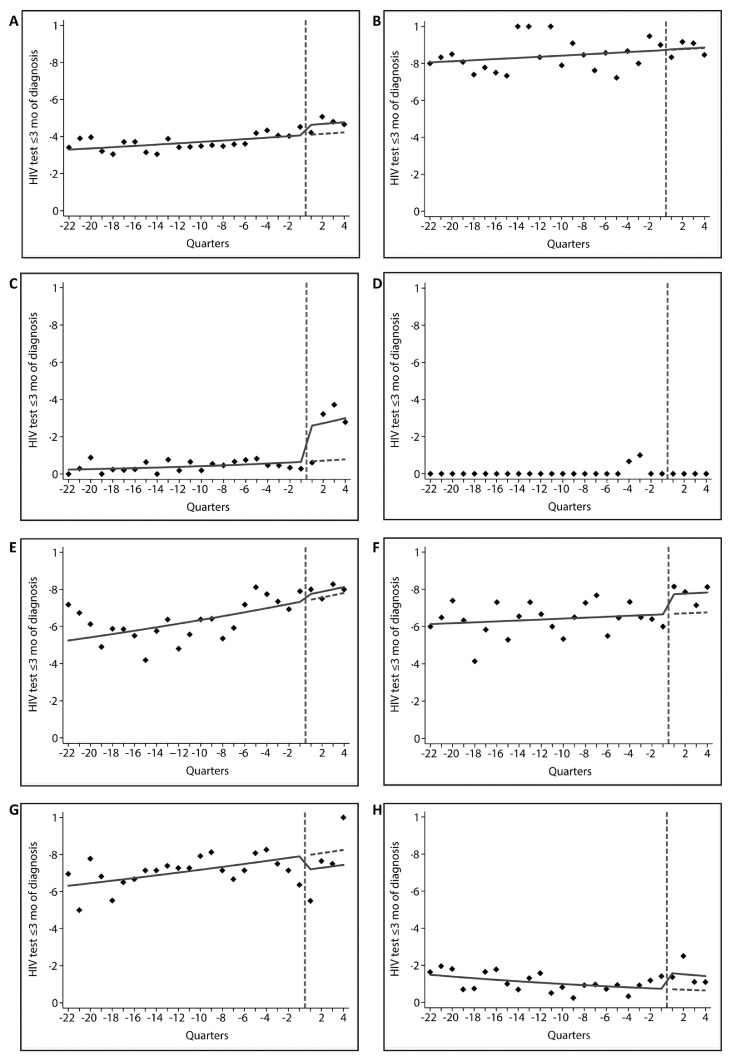
Time-series analysis of the proportion HIV tested within 3 months before or after indicator condition diagnosis overall and by indicator condition. Results are presented overall (Panel A) and by indicator condition: (Panel B) tuberculosis. (Panel C) cervical cancer or intraepithelial neoplasia grade III. (Panel D) vulvar cancer or intraepithelial neoplasia grade III. (Panel E) malignant lymphoma. (Panel F) hepatitis B virus infection. (Panel G) hepatitis C virus infection. (Panel H) peripheral neuropathy. The vertical dotted line represents the transition from the pre-intervention to the intervention period, which consists of a one-year period starting from the start date of the intervention. The diamonds represent the observed proportions tested for HIV within 3 months before or after indicator condition diagnosis by quarter-year period. The solid lines are the trend lines based on unadjusted time-series Poisson regression analysis. The horizontal dashed lines represent the expected trend in the intervention period based on the pre-intervention period. Trends and expected trends could not be obtained for vulvar cancer or intraepithelial neoplasia grade III (Panel D).

Overall, 3068/7986 (38.4%) patients were tested within 3 months before or after IC diagnosis. The proportion HIV tested within 3 months before or after IC diagnosis increased from 2478/6730 (36.8%) in the pre-intervention period to 590/1256 (47.0%) in the intervention period (unadjusted RR 1.13, 95% CI 1.01–1.27, *p*=0.04, Table 4; RR adjusted for patients’ age, sex, SES and pregnant at IC diagnosis: 1.16, 95% CI 1.03–1.30, *p*=0.02, Figure 2, Table 4). For individual IC, significant increases in HIV testing were observed after the intervention among patients with CC/CIN-3 (aRR 3.62, 95% CI 1.93–6.79, *p*<0.001) and PN (aRR 2.27 95% CI 1.48–3.49, *p*=<0.001), but not the other ICs. Stratification by subtypes of ML revealed higher proportions HIV tested in high-grade subtypes compared to low-grade subtypes, but no significant increase in HIV testing using time-series analyses (Table 4). In sensitivity analysis using HIV testing within 3 months after IC diagnosis only, the proportion HIV tested increased from 1506/6730 (22.4%) to 367/1256 (29.2%), aRR 1.33 95% CI 1.14–1.55, *p*=<0.001 (Supplementary Table 4). Results did not change in sensitivity analyses where patients who died within 3 months after IC diagnosis were excluded (overall aRR 1.15, 95% CI 1.02–1.29, *p*=0.02). Stratified by hospital and IC, we noted the same pattern as in the main analysis, except in TB and HCV, where we observed non-significant decreases in proportions tested in three hospitals (Supplementary table 5). In an analysis where we allowed effects to vary by hospital (random effects model), the overall aRR was 1.16 (95% CI 1.03–1.31, *p*=0.01, Supplementary Table 6). For individual IC, the aRRs for CC/CIN-3 (aRR 3.76, 95% CI 2.01–7.04, *p*<0.001) and PN (aRR 2.33, 95% CI 1.52–3.59, *p*<0.001) were also slightly higher in this model.

**Table 4:** Proportion of patients tested for HIV within 3 months before or after indicator condition diagnosis, and unadjusted and adjusted risk ratio's, overall and by indicator condition, Amsterdam region 2015–2021.

	Before intervention (*n*=6730)	After intervention (*n*=1256)	Unadjusted risk ratio (95% CI)	*p* value	Adjusted risk ratio^a^ (95% CI)	*p* value
Overall	2478/6730 (36.8%)	590/1256 (47.0%)	1.13 (1.01–1.27)	0.04	1.16 (1.03–1.30)	0.02
By indicator condition						
Tuberculosis	317/379 (83.6%)	52/59 (88.1%)	1.00 (0.69–1.45)	0.99	1.00 (0.68–1.45)	0.98
Cervical cancer or CIN-3	46/1075 (4.3%)	77/275 (28.0%)	3.81 (2.04–7.11)	<0.001	3.62 (1.93–6.79)	<0.001
Vulvar cancer or VIN-3	2/283 (0.7%)	0/52 (0.0%)	n/a	n/a	n/a	n/a
Malignant lymphoma	1021/1612 (63.3%)	286/361 (79.2%)	1.04 (0.88–1.24)	0.65	1.05 (0.88–1.25)	0.61
Hodgkin's lymphoma	158/228 (69.3%)	32/35 (91.4%)	1.12 (0.70–1.81)	0.64	1.14 (0.71–1.85)	0.58
T-cell lymphoma	111/173 (64.2%)	32/36 (88.9%)	1.16 (0.69–1.94)	0.57	1.13 (0.67–1.89)	0.64
Diffuse large B-cell lymphoma	393/536 (73.3%)	132/150 (88.0%)	1.05 (0.80–1.37)	0.72	1.04 (0.80–1.37)	0.76
Mantle cell lymphoma	65/90 (72.2%)	22/26 (84.6%)	1.11 (0.56–2.19)	0.76	1.21 (0.60–2.46)	0.59
Follicular lymphoma	121/234 (51.7%)	18/37 (48.7%)	0.67 (0.37–1.22)	0.19	0.68 (0.37–1.24)	0.21
Marginal zone/MALT lymphoma	58/133 (43.6%)	21/32 (65.6%)	1.00 (0.50–1.98)	0.99	1.07 (0.53–2.16)	0.85
Burkitt lymphoma	25/28 (89.3%)	4/4 (100.0%)	n/a	n/a	n/a	n/a
Lymphoplasmacytic lymphoma	4/16 (25.0%)	1/1 (100%)	n/a	n/a	n/a	n/a
Non-Hodgkin lymphoma, other	86/174 (49.4%)	24/40 (60·0%)	1.03 (0.57–1.88)	0.91	0.99 (0.54–1.82)	0.98
Hepatitis B virus infection	520/809 (64.3%)	77/99 (77·8%)	1.16 (0.87–1.54)	0.31	1.16 (0.87–1.54)	0.32
Hepatitis C virus infection	351/499 (70.3%)	46/63 (73·0%)	0.90 (0.62–1.31)	0.59	0.90 (0.61–1.33)	0.60
Peripheral neuropathy	221/2,073 (10.7%)	52/347 (15.0%)	2.22 (1.45–3.39)	<0.001	2.27 (1.48–3.49)	<0.001

a Analyses are performed using multivariable models adjusting for confounding patient characteristics sex, age, socio-economic status, and pregnant at time of indicator condition diagnosis. n/a: parameter estimates could not be obtained. CIN-3: Cervical intraepithelial neoplasia grade III. MALT: mucosa-associated lymphoid tissue. VIN-3: vulvar intraepithelial neoplasia grade III.

Of the 3068 patients tested for HIV within 3 months before or after IC diagnosis, 93.4% had been tested in the hospital setting, 2.9% by their general practitioner, 2.7% during antenatal care services, 0.7% at a sexual health clinic, and 0.3% elsewhere. Of patients tested for HIV, 87.5% in the pre-intervention and 91.3% in the intervention period had been tested before initiating treatment for their IC (*p*=0.03). Compared to those not tested for HIV within 3 months before or after IC diagnosis, patients who had been tested were more often male, younger, were of a lower SES category, and more often deceased within 3 months after IC diagnosis (Supplementary Table 7).

### Patients not tested for HIV within 3 months before or after IC diagnosis

In 92 (1.9%) of the 4918 patients who did not receive HIV testing within 3 months before or after IC diagnosis, there was evidence of an HIV test being offered by the treating physician. In 55/92 (59.8%) of these cases, the patient agreed to receiving an HIV test, but the test was ultimately not performed by decision of the physician or patient. In 9/92 (9.8%) of these cases, the patient explicitly refused the HIV test. In the remaining 28/92 (30.4%) cases, it could not be determined whether the test was accepted or not. In 47/4,918 (1.0%) of patients who did not receive HIV testing within 3 months before or after IC diagnosis, the physician had explicitly noted the reason for not offering HIV testing in the EHR, which included negative HIV tests in the past (*n*=38), no perceived HIV risk (*n*=5), or transfer of care to another facility or primary care (*n*=4).

### Percentage HIV positive

Overall, 18/3068 (0.6%) patients tested HIV positive within 3 months before or after IC diagnosis: 17/2478 (0.7%) in the pre-intervention period and 1/590 (0.2%) in the intervention period (*p*=0.23, Supplementary table 8), exceeding the cost-effectiveness threshold for HIV screening of 0.1% (*p*<0.0001). Eight (44.4%) had TB, seven (38.9%) had ML, two (11.1%) had HBV and one (5.6%) had HCV. Of the seven with ML, five had diffuse large B-cell lymphoma, one had Burkitt's lymphoma and one had T-cell lymphoma. Fourteen (77.8%) of 18 patients were male, the median age was 45 years (IQR 34-54), and the majority lived in a low SES postal-code area (10 [55.6%] low, 3 [16.7%] intermediate, 5 [27.8%] high). Most patients (17/18; 94.4%) received their diagnosis at a late stage. Compared to patients testing HIV negative within 3 months before or after IC diagnosis, patients testing HIV positive were younger (mean age 45 years [IQR 34-54] vs. 52 [IQR 37-64] *p*=0.05), more often male (77.8% vs. 58.0% male, *p*=0.10) and more often of lower SES (55.6% vs. 39.6% low, 16.7% vs. 22.3% intermediate and 27.8% vs. 38.1% high SES, *p*=0.42, Supplementary table 8).

### Proportion HIV tested within 6 months before or after IC diagnosis and ever

Overall, 3327/7986 (41.7%) patients were tested within 6 months before or after IC diagnosis. The proportion HIV tested within 6 months before or after IC diagnosis increased from 2707/6730 (40.2%) in the pre-intervention period to 620/1256 (49.4%) in the intervention period (aRR 1.15, 95% CI 1.02–1.28, *p*=0.02). The proportion of patients ever tested for HIV before or up to 6 months after IC diagnosis did not increase significantly (from 3355/6730 [49.9%] to 761/1256 [60.6%]; aRR 1.08, 95% CI 0.98–1.20, *p*=0.14).

## Discussion

This multifaceted intervention resulted in an overall 10.2% absolute increase in IC-guided HIV testing within 3 months before or after IC diagnosis. The overall proportion HIV tested within 3 months before or after IC diagnosis improved significantly following the intervention in this interrupted time-series analysis. The crude proportion HIV tested increased in all ICs except VC/VIN-3, and in all ML subtypes except follicular lymphoma. However, a significant increase in HIV testing was only observed in CC/CIN-3 and PN, the ICs with the lowest pre-intervention proportion HIV tested. HIV testing within 3 months before or after IC diagnosis was still only done in less than half of included patients following the intervention, highlighting persistent missed opportunities for HIV testing.

We observed large variation in HIV testing in the pre-intervention phase. HIV testing was already reasonably high among patients with TB, HCV and high-grade subtypes of ML (i.e., 84%, 70% and 64–89% respectively), but considerable improvement was warranted among patients diagnosed with other ICs. A possible explanation is the lack of routine HIV testing recommendations in specialty guidelines for PN, CC/CIN-3, VC/VIN-3 and several ML subtypes, particularly low-grade ones, while HIV testing is explicitly recommended in TB, HBV and HCV guidelines, as well as some ML subtype guidelines.^14^, ^15^, ^16^, ^17^, ^18^, ^19^, ^20^ This difference in testing recommendations is reflected in our data, where we observed lower crude proportions HIV tested among patients diagnosed with follicular lymphoma and marginal zone lymphoma compared to other lymphomas. Additionally, physician beliefs of the importance of IC-guided HIV testing may have played a role, such as low perceived risk among women and older patients, which could explain the lower proportion tested for HIV in these groups. Patients with VC/VIN-3, the IC with the oldest population, were tested least.

Several specialty departments implemented additional changes at varying times triggered by the intervention, which may have influenced HIV testing (Table 3). While the design of the educational sessions was identical per hospital and specialty, it is therefore challenging to disentangle the direct effect of the intervention versus these varying intervention developments. For example, we saw the largest effect among patients diagnosed with CC/CIN-3, specifically at one university hospital. HIV testing recommendations had been lacking from CC/CIN-3 guidelines prior to the intervention; immediately following the educational meeting, it was added by gynaecologists at this hospital to their local guideline as well as the standard laboratory orders for new patients diagnosed with cervical carcinoma. In the one non-academic teaching hospital where this recommendation was also added to the local guideline, we observed an absolute increase in HIV testing of 10%. In that same hospital, neurologists added HIV testing recommendations to their local PN guidelines following the educational intervention meeting and HIV testing among PN patients increased from 14% to 26%. Although these settings with guideline revisions demonstrated modest increases in HIV testing, such revisions alone might not be sufficient to have a substantial impact on HIV testing.^21^^,^^22^ Possibly, additional intervention strategies to support guideline adaptations would have been more effective, such as adapting the laboratory orders to automatically include an HIV test. The absolute overall increase of 10% in IC-guided HIV testing within 3 months before or after IC diagnosis observed in our data is comparable to that in other multifaceted intervention programmes aiming to improve IC-guided testing for HIV in Europe,^23^^,^^24^ as well as interventions aiming to improve appropriate treatment strategies for other infectious diseases.^25^^,^^26^

Previous studies have determined routine HIV screening is cost-effective in comparable settings where the undiagnosed HIV prevalence is >0.1%.^5^^,^^27^^,^^28^ The percentage that tested HIV positive of 0.6% observed in our study was substantially above this cost-effectiveness threshold, indicating that this strategy to identify undiagnosed people living with HIV is indeed cost-effective. However, although the proportion tested increased, we observed a lower percentage positive after the intervention than before (0.2% vs. 0.7%, respectively), and a percentage positive of 0% in some ICs. Thus, the question arises when this routine HIV testing strategy is no longer cost-effective in our setting. We therefore believe that focusing on ICs with the largest undiagnosed HIV prevalence, including TB, ML and HBV, may be most efficient when working with limited resources for interventions. Additionally, nearly all newly diagnosed people living with HIV in this study received their diagnosis at a late stage, suggesting that while this strategy was cost-effective to identify undiagnosed individuals, it may not be an optimal strategy for early HIV diagnosis.

The low number of patients who explicitly refused HIV testing concurs with findings from other studies reporting that this approach is acceptable for patients diagnosed with ICs^6^^,^^7^; we do not expect that refusal of HIV testing when offered by the physician goes unrecorded in the EHRs. In the majority of cases where no HIV testing was done within 3 months before or after IC diagnosis, no reasoning was reported by the physician, while only in 1% of cases the physician had a justified reason for not testing. As it is likely that physicians will report any conscious deviation from recommended diagnostic approaches in patients’ EHR, among cases where no reasoning was reported, explicit deliberation on HIV testing was probably not done.

The main strength of this study is the large number of included patients per IC, the participation of various types of hospitals (i.e., university, teaching and non-teaching), which increases the generalizability of our findings to other settings in the Netherlands and other low-prevalence, high-income settings. Additionally, using time-series regression to estimate the effect of our intervention allowed us to correct for trends in HIV testing that would have otherwise been disregarded in a study design comparing outcomes before and after a given intervention, possibly leading to an overestimation of the intervention's effect.^29^ Third, we employed feasible, low-cost elements in our local interventions that were tailored to hospital and specialty department. Additional opportunities for implementation were identified during discussion at several educational meetings. Physicians were actively involved in the local development of strategies and implementation. Consequently, we ensured that the intervention was appropriate and relevant for each setting specifically, and therefore more likely to be impactful.^30^

A considerable limitation of our study is the short follow-up time after the intervention. While the intervention phase launched in all sites on the same date, implementation of various site-specific intervention developments was more outspread, and the effect of some developments might therefore not be apparent in our data if their implementation was finalised towards the end of the phase. Due to the short follow-up time, we can also not report on the sustainability of the effect of our interventions. However, it is likely that structural intervention developments, such as adding HIV testing to orders, are likely to yield sustained improvement in HIV testing.^9^ Second, as we collected our data from patient EHRs, certain data such as migration background was unavailable and could not be accounted for in analyses. Additionally, reporting bias might have occurred if patients were tested for HIV outside of the hospital setting and this was not reported in the EHR. However, as physicians are expected to report any reasoning for deliberately deviating from recommended practice in patient EHRs, we would then have expected to find more reports of HIV tests done elsewhere in this case, but only 7% of tests took place outside the hospital setting. Finally, The COVID-19 pandemic may have negatively impacted the effect of our intervention. As the intervention phase was conducted during this pandemic, while restrictions were imposed by the Dutch government, several educational meetings were conducted through video-conferencing. This, and the increased strain on healthcare workers during this time, might have reduced the effect of the meetings. However, attendance at the meetings was not impacted as educational meetings were still routinely attended during this period.

## Conclusion

The multifaceted intervention increased IC-guided HIV testing, but its effect varied by IC, possibly due to variations in implemented developments as well as a short follow-up period. Our study confirmed the cost-effectiveness of this testing strategy to identify undiagnosed people living with HIV, underlining its importance in contributing to end HIV transmission.

## Contributors

SJB, MFSL, JEAMB and SEG designed the study. SEG and JEAMB acquired funding. SJB recruited patients, collected data, supervised the junior researchers collecting data, and wrote the first and final draft of the manuscript. MFSL and AB collaborated in the statistical analysis. SJB performed all data cleaning and analyses, which was all subsequently checked by MFSL. UD was involved in the design of the questionnaire. KB, KS, JB, NB and SEG supported local implementation of the intervention and data collection. All authors had access to the data used in this study. All authors interpreted the data and revised the manuscript critically for important intellectual content. All authors read and approved the final manuscript.

## Data sharing statement

Data collected for this study will be made available upon reasonable request directed to the principal investigator, Prof Suzanne E. Geerlings (s.e.geerlings@amsterdamumc.nl) after completing a data sharing agreement.

## Declaration of interests

Dr. Bogers has nothing to disclose. Dr. Schim van der Loeff has nothing to disclose. Dr. Boyd reports grants or contracts: ANRS, ZonMW and Participation on the Data Safety Monitoring Board or Advisory Board: Amsterdam University Medical Centers, Inserm. Dr. Davidovich has nothing to disclose. Dr. van der Valk reports grants or contracts: ViiV Healthcare, Gilead Sciences and Participation on the Data Safety Monitoring Board or Advisory Board: Viiv Healthcare, Gilead Sciences, MSD. Reimbursement paid to institution, and Leadership or fiduciary role in other board, society, committee or advocacy group, paid or unpaid: Member EACS ART and comorbidities guideline committee. Dr. Brinkman has nothing to disclose. Dr. Sigaloff has nothing to disclose. Dr. Branger has nothing to disclose. Dr. Bokhizzou has nothing to disclose. Dr. de Bree has nothing to disclose. Dr. Reiss reports grants or contracts: Gilead Sciences; ViiV Healthcare; Merck: Investigator-initiated study grants to institution and Participation on the Data Safety Monitoring Board or Advisory Board: Gilead Sciences; ViiV Healthcare; Merck: Honoraria for scientific advisory board participation paid to institution. Dr. van Bergen has nothing to disclose. Dr. Geerlings has nothing to disclose.

## HIV Transmission Elimination AMsterdam (H-TEAM) Initiative

**The H-TEAM members are**

T. van Benthem^1^, D. Bons^2^, G.J. de Bree^3;4^, P. Brokx^5^, U. Davidovich^1;6^, F. Deug^7^, S.E. Geerlings^4^, M. Heidenrijk^3^, E. Hoornenborg^1^, M. Prins^1;4^, P. Reiss^3;5^, A. van Sighem^8^, M. van der Valk^4;8^, J. de Wit^9^, W. Zuilhof^7^.

**H-TEAM Project Management**

N. Schat^3^, D. Smith.

**H-TEAM additional collaborators**

M. van Agtmael^10^, J. Ananworanich^11^, D. van de Beek^12^, G.E.L. van den Berk^13^, D. Bezemer^8^, A. van Bijnen^7^, J.P. Bil^1^, W.L. Blok^12^, S.J. Bogers^4^, M. Bomers^10^, A. Boyd^1;8^, W. Brokking^14^, D. Burger^15^, K. Brinkman^13^, N. Brinkman^13^, M. de Bruin^16^, S. Bruisten^1^, L. Coyer^1^, R. van Crevel^17^, M. Dijkstra^1^, Y.T. van Duijnhoven^1^, A. van Eeden^14^, L. Elsenburg^14^, M.A.M. van den Elshout^1^, E. Ersan^18^, P.E.V. Felipa^1^, T.B.H. Geijtenbeek^19^, J. van Gool^1^, A. Goorhuis^4^, M. Groot^14^, C.A. Hankins^3^, A. Heijnen^20;21^, M.M.J Hillebregt^8^, M. Hommenga^1^, J.W. Hovius^4^, Y. Janssen^22^, K. de Jong^1^, V. Jongen^1^, N.A. Kootstra^23^, R.A. Koup^24^, F.P. Kroon^25^, T.J.W. van de Laar^26;27^, F. Lauw^28^, M.M. van Leeuwen^5^, K. Lettinga^29^, I. Linde^1^, D.S.E. Loomans^1^, I.M. van der Lubben^1^, J.T. van der Meer^4^, T. Mouhebati^7^, B.J. Mulder^1^, J. Mulder^30^, F.J. Nellen^4^, A. Nijsters^7^, H. Nobel^4^, E.L.M. Op de Coul^31^, E. Peters^10^, I.S. Peters^1^, T. van der Poll^4^, O. Ratmann^32^, C. Rokx^33^, M.F. Schim van der Loeff^1;34^, W.E.M. Schouten^13^, J. Schouten^1^, J. Veenstra^29^, A. Verbon^33^, F. Verdult^5^, J. de Vocht^10^, H.J. de Vries^1;34;35^, S. Vrouenraets^29^, M. van Vugt^4^, W.J. Wiersinga^4^, F.W. Wit^4;6^, L.R. Woittiez^4^, S. Zaheri^8^, P. Zantkuijl^7^, A. Żakowicz^36^, M.C. van Zelm^37^, H.M.L. Zimmermann^1^

**Affiliations:**

^1^Department of Infectious Diseases, Public Health Service of Amsterdam, Amsterdam, the Netherlands

^2^Trans United Europe, Amsterdam, The Netherlands

^3^Department of Global Health, Amsterdam UMC – location AMC, and Amsterdam Institute for Global Health and Development, Amsterdam, the Netherlands

^4^Department of Internal Medicine, Division of Infectious Diseases, Amsterdam UMC – location AMC, Amsterdam, the Netherlands

^5^Dutch Association of PLHIV, Amsterdam, the Netherlands

^6^Department of Social Psychology, University of Amsterdam, Amsterdam, the Netherlands

^7^Soa Aids Nederland, Amsterdam, the Netherlands

^8^Stichting HIV Monitoring, Amsterdam, the Netherlands

^9^Department of Interdisciplinary Social Science: Public Health, Utrecht University, Utrecht, the Netherlands

^10^Department of Internal Medicine, Amsterdam UMC – location VUMC, Amsterdam, the Netherlands

^11^US Military HIV Research Program and the Henry M. Jackson Foundation for the Advancement of Military Medicine, Bethesda, United States

^12^Center of Infection and Immunity Amsterdam (CINIMA), Department of Neurology, Amsterdam UMC – location AMC, Amsterdam, the Netherlands

^13^Department of internal medicine, OLVG – location East, Amsterdam, the Netherlands

^14^DC Klinieken, Amsterdam, the Netherlands

^15^Department of Pharmacy, Radboud University Nijmegen Medical Center, Nijmegen, the Netherlands

^16^Aberdeen Health Psychology Group, Institute of Applied Health Sciences, University of Aberdeen, Aberdeen, United Kingdom

^17^Department of Internal Medicine, Radboud University Nijmegen Medical Center, Nijmegen, the Netherlands

^18^Department of General Practice, Amsterdam UMC – location AMC, University of Amsterdam, Amsterdam, the Netherlands

^19^Laboratory of Experimental Immunology, Amsterdam UMC – location AMC Amsterdam, the Netherlands

^20^Sexology Center Amsterdam, Amsterdam, the Netherlands

^21^GP practice Heijnen & de Meij, Amsterdam, the Netherlands

^22^Primary Care Amsterdam and Almere (Elaa), Amsterdam, the Netherlands

^23^Laboratory for Viral Immune Pathogenesis, Amsterdam UMC – location AMC Amsterdam, the Netherlands

^24^Immunology Laboratory, Vaccine Research Center, National Institute of Allergy and Infectious Diseases, National Institutes of Health, Rockville, Maryland, USA

^25^Department of Infectious Diseases, Leiden University Medical Center, Leiden, the Netherlands

^26^Department of Medical Microbiology, OLVG, Amsterdam, the Netherlands

^27^Department of Donor Medicine Research, Laboratory of Blood-borne Infections, Sanquin Research, Amsterdam, the Netherlands

^28^Department of Internal Medicine, Medical Center Jan van Goyen, Amsterdam, the Netherlands

^29^Department of Internal Medicine, OLVG – location West, Amsterdam, the Netherlands

^30^Department of Internal Medicine, Slotervaart Hospital (former), Amsterdam, the Netherlands

^31^Epidemiology and Surveillance Unit, Center for Infectious Disease Control, National Institute of Public Health and the Environment, the Netherlands

^32^School of Public Health, Faculty of Medicine, Imperial College London, London, United Kingdom

^33^Department of Internal Medicine and Infectious Diseases, Erasmus Medical Center, Rotterdam, the Netherlands

^34^Center for Infection and Immunology, Amsterdam (CINIMA), Amsterdam UMC – location AMC, University of Amsterdam, Amsterdam, the Netherlands

^35^Department of Dermatology, Amsterdam UMC – location AMC, University of Amsterdam, Amsterdam, the Netherlands

^36^AIDS Healthcare Foundation, Amsterdam, the Netherlands

^37^Department of Virology, Erasmus Medical Center, Rotterdam, the Netherlands

## Appendix B Supplementary materials

Image, application
1
