# Supplementary material for: Improving indicator-condition guided testing for HIV in the hospital setting (PROTEST 2·0): A multicenter, interrupted time-series analysis
Source: Lancet Reg Health Eur. 2022 Oct 7;23:100515. doi: 10.1016/j.lanepe.2022.100515 (PMC9558045; doi:10.1016/j.lanepe.2022.100515)
Supplement: Supplementary file 1 [file mmc1.docx]

Supplementary files to:

TITLE:

Improving indicator-condition guided testing for HIV in the hospital setting (PROTEST 2·0): a multicenter, interrupted time-series analysis.

**AUTHORS:**

Saskia J. Bogers^a-c^, Maarten F. Schim van der Loeff^a, b, d^, Anders Boyd^b,d,e^, Udi Davidovich^d^, Marc van der Valk^a,b,e^, Kees Brinkman^f^, Kim Sigaloff^b,g^, Judith Branger^h^, Nejma Bokhizzou^i^, Godelieve J. de Bree^a^, Peter Reiss^a,j,k^, Jan E.A.M. van Bergen^l,m^ and Suzanne E. Geerlings^a-c^, on behalf of the HIV Transmission Elimination AMsterdam (H-TEAM) Initiative

**AUTHOR AFFILIATIONS**

^a^ Amsterdam UMC location University of Amsterdam, Internal Medicine, Meibergdreef 9, Amsterdam, The Netherlands

^b^ Amsterdam Institute for Infection and Immunity, Infectious diseases, Amsterdam, the Netherlands.

^c^ Amsterdam Public Health Research Institute, Quality of Care, Amsterdam, the Netherlands.

^d^ Department of Infectious Diseases, Public Health Service of Amsterdam, Amsterdam, the Netherlands.

^e^ Stichting hiv monitoring, Amsterdam, the Netherlands.

^f^ Department of Internal Medicine, Onze Lieve Vrouwe Gasthuis, Amsterdam, the Netherlands.

^g^ Amsterdam UMC location Vrije Universiteit Amsterdam, Internal Medicine, De Boelelaan 1117, Amsterdam, The Netherlands

^h^ Department of Internal Medicine, Flevoziekenhuis, Almere, the Netherlands.

^i^ Department of Internal Medicine, BovenIJ ziekenhuis, Amsterdam, the Netherlands.

^j^ Amsterdam institute for Global Health and Development, Amsterdam, the Netherlands.

^k^ Amsterdam UMC location University of Amsterdam, Global Health, Meibergdreef 9, Amsterdam, The Netherlands

^l^ Amsterdam UMC location University of Amsterdam, General Practice, Meibergdreef 9, Amsterdam, The Netherlands

^m^ STI AIDS Netherlands, Amsterdam, the Netherlands.

**CORRESPONDING AUTHOR**

S.J. Bogers, MD

Amsterdam UMC location University of Amsterdam, Internal Medicine, room D3-226, Meibergdreef 9, 1105 AZ, Amsterdam, Amsterdam, The Netherlands.

Email: [s.j.bogers@amsterdamumc.nl](mailto:s.j.bogers@amsterdamumc.nl). Phone: +316-129-178-80.

Table of content:

| - **Table 1:** Revised Standards for Quality Improvement Reporting Excellence (SQUIRE 2·0) checklist | Page 3-4 |
| --- | --- |
| - **Table 2**: Indicator-condition specific inclusion and exclusion criteria | Page 5 |
| - **Table 3**: Data collection form | Page 6-7 |
| - **Table 4:** Proportion of patients tested for HIV within 3 months after indicator condition diagnosis, and unadjusted and adjusted risk ratio’s, overall and by indicator condition, Amsterdam region 2015-2021. | Page 8 |
| - **Table 5:** Proportions of patients tested for HIV within 3 months before or after indicator condition diagnosis, and unadjusted and adjusted risk ratio’s, by hospital and indicator condition, Amsterdam region 2015-2021. | Page 9-10 |
| - **Table 6**: Proportions tested for HIV within 3 months before or after indicator condition diagnosis, and risk ratio, overall and by indicator condition - Random effects model accounting for variability by hospital. Amsterdam region, 2015-2021. | Page 11 |
| - **Table 7:** Characteristics of patients tested and not tested for HIV within 3 months before or after indicator condition diagnosis in five hospitals in the region of Amsterdam, 2015-2021 | Page 12 |
| - **Table 8:** Characteristics of patients testing HIV positive versus HIV negative within 3 months before or after indicator condition diagnosis in five hospitals in the region of Amsterdam, 2015-2021 | Page 13 |

Supplementary table 1: Revised Standards for Quality Improvement Reporting Excellence (SQUIRE 2·0) checklist

| **Text Section and Item Name** | **Section or Item Description** | **Page reported** |
| --- | --- | --- |
| **Title and Abstract** |  |  |
| **1. Title** | Indicate that the manuscript concerns an initiative to improve healthcare (broadly defined to include the quality, safety, effectiveness, patient-centeredness, timeliness, cost, efficiency, and equity of healthcare) | 1 |
| **2. Abstract** | a. Provide adequate information to aid in searching and indexing  b. Summarize all key information from various sections of the text using the abstract format of the intended publication or a structured summary such as: background, local problem, methods, interventions, results, conclusions | 2 |
| **Introduction** | *Why did you start?* |  |
| **3. Problem Description** | Nature and significance of the local problem | 4 |
| **4. Available knowledge** | Summary of what is currently known about the problem, including relevant previous studies | 4 |
| **5. Rationale** | Informal or formal frameworks, models, concepts, and/or theories used to explain the problem, any reasons or assumptions that were used to develop the intervention(s), and reasons why the intervention(s) was expected to work | 4 |
| **6. Specific aims** | Purpose of the project and of this report | 4-5 |
| **Methods** | *What did you do?* |  |
| **7. Context** | Contextual elements considered important at the outset of introducing the intervention(s) | 5-6 |
| **8. Intervention(s)** | a. Description of the intervention(s) in sufficient detail that others could reproduce it  b. Specifics of the team involved in the work | 5, tables 1 & 3 |
| **9. Study of the Intervention(s)** | a. Approach chosen for assessing the impact of the intervention(s)  b. Approach used to establish whether the observed outcomes were due to the intervention(s) | 6-8 |
| **10. Measures** | a. Measures chosen for studying processes and outcomes of the intervention(s), including rationale for choosing them, their operational definitions, and their validity and reliability  b. Description of the approach to the ongoing assessment of contextual elements that contributed to the success, failure, efficiency, and cost  c. Methods employed for assessing completeness and accuracy of data | 6-7, supplementary tables 2-3 |
| **11. Analysis** | a. Qualitative and quantitative methods used to draw inferences from the data  b. Methods for understanding variation within the data, including the effects of time as a variable | 7-8 |
| **12. Ethical Considerations** | Ethical aspects of implementing and studying the intervention(s) and how they were addressed, including, but not limited to, formal ethics review and potential conflict(s) of interest | 8 |
| **Results** | *What did you find?* |  |
| **13. Results** | a. Initial steps of the intervention(s) and their evolution over time (*e.g.*, time-line diagram, flow chart, or table), including modifications made to the intervention during the project  b. Details of the process measures and outcome  c. Contextual elements that interacted with the intervention(s)  d. Observed associations between outcomes, interventions, and relevant contextual elements  e. Unintended consequences such as unexpected benefits, problems, failures, or costs associated with the intervention(s).  f. Details about missing data | 9-11,  tables 1-4, supplementary tables 4-8 |
| **Discussion** | *What does it mean?* |  |
| **14. Summary** | a. Key findings, including relevance to the rationale and specific aims  b. Particular strengths of the project | 12-14 |
| **15. Interpretation** | a. Nature of the association between the intervention(s) and the  outcomes  b. Comparison of results with findings from other publications  c. Impact of the project on people and systems  d. Reasons for any differences between observed and anticipated outcomes, including the influence of context  e. Costs and strategic trade-offs, including opportunity costs | 12-15 |
| **16. Limitations** | a. Limits to the generalizability of the work  b. Factors that might have limited internal validity such as confounding, bias, or imprecision in the design, methods, measurement, or analysis  c. Efforts made to minimize and adjust for limitations | 14-15 |
| **17. Conclusions** | a. Usefulness of the work  b. Sustainability  c. Potential for spread to other contexts  d. Implications for practice and for further study in the field  e. Suggested next steps | 15, 16-17 |
| **Other information** |  |  |
| **18. Funding** | Sources of funding that supported this work. Role, if any, of the funding organization in the design, implementation, interpretation, and reporting | 8 |

Supplementary table 2: Indicator-condition specific inclusion and exclusion criteria

| Tuberculosis | Patients with latent *M. tuberculosis* infection, but no tuberculosis disease, were excluded. |
| --- | --- |
| Cervical cancer or CIN-3 | Patients without biopsy-confirmed grade III intraepithelial neoplasia or carcinoma (invasive or non-invasive) were excluded. |
| Vulvar cancer or VIN-3 | Patients without biopsy-confirmed grade III intraepithelial neoplasia or carcinoma (invasive or non-invasive) were excluded. |
| Malignant lymphoma | All types of malignant lymphoma, including all subtypes of Hodgkin’s lymphoma and non-Hodgkin lymphoma were included. |
| Hepatitis B virus infection | Both acute and chronic, and active and inactive hepatitis B virus infection cases were included. Hepatitis B virus infection cases with and without detectable serum replication were included. |
| Hepatitis C virus infection | Both acute and chronic, and active and inactive hepatitis C virus infection cases were included. |
| Peripheral neuropathy | Patients with known diabetes mellitus before presentation and patients for whom no diagnostic laboratory workup was indicated/performed were excluded. |

CIN-3: Cervical intraepithelial neoplasia grade III. IC: Indicator condition. VIN-3: vulvar intraepithelial neoplasia grade III.

Supplementary table 3: Data collection form

*Content of the data collection form used for the collection of data from the electronic health records of eligible patients*

|  | ***Form question*** | ***Options*** | ***Quality check*** |
| --- | --- | --- | --- |
|  | ***Inclusion*** | | |
| *1.1* | *Is the patient diagnosed with one of the selected IC?* | *Yes/no* | *Exclusion message appears when ‘no’ is selected* |
| *1.2* | *Is the patient aged ≥18 years at diagnosis of selected IC?* | *Yes/no* | *Exclusion message appears when ‘no’ is selected* |
| *1.3* | *Is the patient known HIV positive prior to the diagnostic process for selected IC (i.e. fist presentation with/for the selected IC)?* | *Yes/no* | *Exclusion message appears when ‘no’ is selected* |
|  | ***Patient characteristics*** | | |
| *2.1* | *Patient year of birth* | *4-digit field* | *Lower/upper limit: 1900 - 2004* |
| *2.2* | *Year of IC diagnosis* | *4-digit field* | *Lower/upper limit: 2015 - 2021* |
| *2.3* | *Patient sex* | *Male/Female/Other* | *n/a* |
| *2.4* | *Is patient pregnant at IC diagnosis?* | *Yes/No* | *Shown if 2.3 = female* |
| *2.5* | *Socio-economic status of postal-code area of patient* | *3-digit field* | *Values ranging from -7.78 to 2.82 based on patient’s 4-digit postal-code. Values were subsequently recoded into low/intermediate or high socio-economic status based on national tertile segments.* |
| *2.6* | *Is the patient deceased?* | *Yes/No* | *n/a* |
| *2.7* | *Date of passing* | *Date field* | *Shown if 2.6 = Yes* |
|  | ***Indicator condition*** | | |
| *3.1* | *Indicator condition diagnosed* | *Tuberculosis*  *Cervical carcinoma/CIN-3*  *Vulvar carcinoma/VIN-3*  *Malignant lymphoma*  *Hepatitis B virus infection*  *Hepatitis C virus infection*  *Peripheral neuropathy* | *n/a* |
| *3.2* | *Date of IC diagnosis* | *Date field* | *n/a* |
| *3.3* | *Type of lymphoma* | *Text field* | *Shown if 3.1 = malignant lymphoma* |
| *3.4* | *Is the patient newly diagnosed?* | *Yes/No* | *Shown if 3.1 = malignant lymphoma* |
| *3.5* | *Newly diagnosed lymphoma* | *Requiring immediate treatment*  *Required delayed treatment*  *Not requiring treatment* | *Shown if 3.4 = Yes* |
| *3.6* | *Not newly diagnosed lymphoma* | *Progressive, requiring treatment*  *Recurrent, after remission*  *Second opinion*  *Transfer from another hospital* | *Shown if 3.4 = No* |
| *3.7* | *Is the patient treated for the IC?* | *Yes/No* | *n/a* |
| *3.8* | *Date of IC treatment initiation* | *Date field* | *Shown if 3.7 = Yes* |
| *3.9* | *Date of IC treatment abstention (i.e. no treatment was initiated)* | *Date field* | *Shown if 3.7 = No* |
| *3.10* | *Remarks on treatment abstention* | *Text field* | *Shown if 3.7 = No* |
|  | ***HIV testing*** | | |
| *4.1* | *Was an HIV test ever performed?* | *Yes/Yes, as screening during pregnancy/No* | *n/a* |
| *4.2* | *Was an HIV test performed within 3 months before or after IC diagnosis?* | *Yes/Yes, as screening during pregnancy/No* | *Shown if 4.1 = Yes* |
| *4.3* | *When was this test done?* | *Date field* | *Shown if 4.2 = Yes* |
| *4.4* | *Where was this test done?* | *Hospital/General practitioner/sexual health clinic/Midwife/Self-test/Community testing/Other* | *Shown if 4.2 = Yes* |
| *4.5* | *What was the HIV test result?* | *Positive/Negative* | *Shown if 4.2 = Yes* |
| *4.6* | *Was a CD4 count determined after HIV diagnosis* | *Yes/No* | *Shown if 4.5 = Positive* |
| *4.7* | *Date of CD4 count determined* | *Date field* | *Shown if 3.6 = Yes* |
| *4.8* | *Value of CD4 count determined (unit cells/mm^3^)* | *4-digit field* | *Shown if 3.6 = Yes; Lower/upper limit: 0-1000* |
| *4.9* | *Was an HIV test performed more than 3 months, but less than 6 months before or after IC diagnosis?* | *Yes/Yes, as screening during pregnancy/No* | *Shown if 4.1 = Yes* |
| *4.10* | *When was this test done?* | *Date field* | *Shown if 4.9 = Yes* |
| *4.11* | *Where was this test done?* | *Hospital/General practitioner/sexual health clinic/Midwife/Self-test/Community testing/Other* | *Shown if 4.9 = Yes* |
| *4.12* | *What was the HIV test result?* | *Positive/Negative* | *Shown if 4.9 = Yes* |
| *4.13* | *Was a CD4 count determined after HIV diagnosis* | *Yes/No* | *Shown if 4.12 = Positive* |
| *4.14* | *Date of CD4 count determined* | *Date field* | *Shown if 3.13 = Yes* |
| *4.15* | *Value of CD4 count determined (unit cells/mm^3^)* | *4-digit field* | *Shown if 3.13 = Yes; Lower/upper limit: 0-1000* |
| *4.16* | *Was an HIV test performed >6 months before or after IC diagnosis?* | *Yes/Yes, as screening during pregnancy/No* | *Shown if 4.1 = Yes* |
| *4.17* | *When was this test done?* | *Date field* | *Shown if 4.16 = Yes* |
| *4.18* | *Where was this test done?* | *Hospital/General practitioner/sexual health clinic/Midwife/Self-test/Community testing/Other* | *Shown if 4.16 = Yes* |
| *4.19* | *What was the HIV test result?* | *Positive/Negative* | *Shown if 4.16 = Yes* |
| *4.20* | *Was a CD4 count determined after HIV diagnosis* | *Yes/No* | *Shown if 4.19 = Positive* |
| *4.21* | *Date of CD4 count determined* | *Date field* | *Shown if 3.20 = Yes* |
| *4.22* | *Value of CD4 count determined (unit cells/mm^3^)* | *4-digit field* | *Shown if 3.20 = Yes; Upper/lower limit: 0-1000* |
| *4.23* | *Was an HIV test offered by the physician at IC diagnosis?* | *Yes/No* | *Shown if 4.1 ,or 4.2 and 4.9 = No* |
| *4.24* | *Did the patient agree to the HIV test?* | *Yes/No* | *Shown if 4.23 = Yes* |
| *4.25* | *Comment on not testing* | *Text field* | *Shown if 4.23 = Yes* |
| *4.26* | *Was a reason for not offering HIV testing reported by the physician?* | *Yes/No* | *Shown if 4.23 = No* |
| *4.27* | *Comment on not offering* | *Text field* | *Shown if 4.23 = No* |
| *4.28* | *Other remarks* | *Text field* | *n/a* |

*CIN-3: cervical intraepithelial neoplasia grade 3. IC: indicator condition. n/a: not applicable. VIN-3: vulvar intraepithelial neoplasia grade 3.*

Supplementary table 4: Proportion of patients tested for HIV within 3 months after indicator condition diagnosis, and unadjusted and adjusted risk ratio’s, overall and by indicator condition, Amsterdam region 2015-2021.

|  | Before intervention (n=6,730) | After intervention (n=1,256) | Unadjusted risk ratio  (95% CI) | *p* value | Adjusted risk ratio*  (95% CI) | *p* value |
| --- | --- | --- | --- | --- | --- | --- |
| Overall | 1,506/6,730 (22·4%) | 367/1,256 (29·2%) | 1·32 (1·13-1·53) | <0·001 | 1·33 (1·14-1·55) | <0·001 |
| By indicator condition | | | | | | |
| Tuberculosis | 183/379 (48·3%) | 32/59 (54·2%) | 1·12 (0·69-1·81) | 0·65 | 1·06 (0·65-1·73) | 0·82 |
| Cervical cancer or CIN-3 | 36/1,075 (3·4%) | 66/275 (24·0%) | 4·35 (2·15-8·79) | <0·001 | 4·09 (2·01-8·29) | <0·001 |
| Vulvar cancer or VIN-3 | 1/283 (0·4%) | 0/52 (0·0%) | n/a | n/a | n/a | n/a |
| Malignant lymphoma | 632/1,612 (39·2%) | 190/361 (52·6%) | 1·03 (0·83-1·28) | 0·80 | 1·03 (0·83-1·28) | 0·80 |
| Hodgkin's lymphoma | 76/228 (33·3%) | 19/35 (54·3%) | 1·21 (0·63-2·31) | 0·56 | 1·19 (0·62-2·30) | 0·60 |
| T-cell lymphoma | 75/173 (43·4%) | 19/36 (52·8%) | 0·84 (0·45-1·59) | 0·60 | 0·83 (0·44-1·56) | 0·56 |
| Diffuse large B-cell lymphoma | 271/536 (50·6%) | 94/150 (62·7%) | 1·07 (0·78-1·47) | 0·68 | 1·07 (0·77-1·48) | 0·69 |
| Mantle cell lymphoma | 53/90 (58·9%) | 15/26 (57·7%) | 0·79 (0·37-1·71) | 0·55 | 0·87 (0·39-1·95) | 0·74 |
| Follicular lymphoma | 66/234 (28·2%) | 12/37 (32·4%) | 0·70 (0·33-1·48) | 0·35 | 0·69 (0·32-1·46) | 0·33 |
| Marginal zone/MALT lymphoma | 33/133 (24·8%) | 17/32 (53·1%) | 1·62 (0·68-3·87) | 0·28 | 1·62 (0·67-3·91) | 0·28 |
| Burkitt lymphoma | 18/28 (64·3%) | 2/4 (50·0%) | 0·98 (0·18-5·41) | 0·98 | 0·85 (0·15-4·73) | 0·85 |
| Lymphoplasmacytic lymphoma | 2/16 (12·5%) | 0/1 (0·0%) | n/a | n/a | n/a | n/a |
| Non-Hodgkin lymphoma, other | 38/174 (21·8%) | 12/40 (30·0%) | 0·90 (0·38-2·13) | 0·81 | 0·84 (0·35-2·02) | 0·70 |
| Hepatitis B virus infection | 267/809 (33·0%) | 23/99 (23·2%) | 1·10 (0·67-1·79) | 0·71 | 1·11 (0·68-1·81) | 0·68 |
| Hepatitis C virus infection | 231/499 (46·3%) | 18/63 (28·6%) | 0·77 (0·44-1·34) | 0·35 | 0·76 (0·43-1·35) | 0·35 |
| Peripheral neuropathy | 156/2,073 (7·5%) | 38/347 (11·0%) | 2·39 (1·44-3·96) | 0·001 | 2·43 (1·46-4·05) | 0·001 |

*Analyses are performed using multivariable models adjusting for confounding patient characteristics sex, age, socio-economic status, and pregnant at time of indicator condition diagnosis. CIN-3: Cervical intraepithelial neoplasia grade III. n/a: parameter estimates could not be obtained. MALT: mucosa-associated lymphoid tissue. VIN-3: vulvar intraepithelial neoplasia grade III.

Supplementary table 5: Proportions of patients tested for HIV within 3 months before or after indicator condition diagnosis, and unadjusted and adjusted risk ratio’s, by hospital and indicator condition, Amsterdam region 2015-2021.

|  | Before intervention | After  intervention | Unadjusted  risk ratio*  (95% CI) | *p* value | Adjusted  risk ratio**  (95% CI) | *p* value |
| --- | --- | --- | --- | --- | --- | --- |
| University hospital 1 | **n= 2,945** | **n=361** |  |  |  |  |
| Tuberculosis | 46/61 (75·4%) | 6/9 (66·7%) | 0·96 (0·35-2·65) | 0·94 | n/a | n/a |
| Cervical cancer or CIN-3 | 34/596 (5·7%) | 65/114 (57·0%) | 4·32 (2·11-8·86) | <0·001 | 4·29 (2·10-8·80) | <0·001 |
| Vulvar cancer or VIN-3 | 1/226 (0·4%) | 0/38 (0·0%) | n/a | n/a | n/a | n/a |
| Malignant lymphoma | 297/473 (62·8%) | 54/69 (78·3%) | 1·09 (0·76-1·58) | 0·63 | 1·15 (0·79-1·66) | 0·47 |
| Hepatitis B virus infection | 155/217 (71·4%) | 11/13 (84·6%) | 1·01 (0·51-2·04) | 0·97 | 1·03 (0·51-2·07) | 0·94 |
| Hepatitis C virus infection | 109/155 (70·3%) | 6/6 (100·0%) | n/a | n/a | n/a | n/a |
| Peripheral neuropathy | 157/1,217 (12·9%) | 16/112 (14·3%) | 2·00 (1·04-3·86) | 0·04 | 2·17 (1·12-4·21) | 0·02 |
| University hospital 2 | **n=919** | **n=164** |  |  |  |  |
| Tuberculosis | 40/51 (78·4%) | 5/5 (100·0%) | 1·08 (0·34-3·43) | 0·90 | n/a | n/a |
| Cervical cancer or CIN-3 | 1/52 (1·9%) | 0/1 (0·0%) | n/a | n/a | n/a | n/a |
| Vulvar cancer or VIN-3 | 0/3 (0·0%) | 0/0 (0·0%) | n/a | n/a | n/a | n/a |
| Malignant lymphoma | 293/471 (62·2%) | 92/111 (82·9%) | 0·96 (0·70-1·31) | 0·80 | 0·96 (0·70-1·31) | 0·79 |
| Hepatitis B virus infection | 83/131 (63·4%) | 11/12 (91·7%) | 1·90 (0·85-4·21) | 0·12 | 1·82 (0·81-4·10) | 0·15 |
| Hepatitis C virus infection | 50/77 (64·9%) | 5/8 (62·5%) | 1·06 (0·36-3·12) | 0·92 | n/a | n/a |
| Peripheral neuropathy | 6/134 (4·5%) | 4/27 (14·8%) | 4·72 (0·55-40·53) | 0·16 | n/a | n/a |
| Teaching hospital 1 | **n=1,531** | **n=360** |  |  |  |  |
| Tuberculosis | 178/205 (86·8%) | 26/28 (92·9%) | 0·93 (0·56-1·56) | 0·79 | n/a | n/a |
| Cervical cancer or CIN-3 | 4/172 (2·3%) | 5/43 (11·6%) | 0·69 (0·08-6·11) | 0·74 | n/a | n/a |
| Vulvar cancer or VIN-3 | 1/27 (3·7%) | 0/9 (0·0%) | n/a | n/a | n/a | n/a |
| Malignant lymphoma | 259/352 (73·6%) | 83/109 (76·1%) | 0·92 (0·66-1·28) | 0·63 | 0·92 (0·66-1·29) | 0·64 |
| Hepatitis B virus infection | 208/321 (64·8%) | 47/62 (75·8%) | 0·98 (0·67-1·43) | 0·92 | 0·96 (0·65-1·41) | 0·84 |
| Hepatitis C virus infection | 152/210 (72·4%) | 32/43 (74·4%) | 0·88 (0·54-1·44) | 0·61 | 0·94 (0·55-1·59) | 0·81 |
| Peripheral neuropathy | 33/244 (13·5%) | 17/66 (25·8%) | 1·49 (0·65-3·42) | 0·35 | 1·54 (0·64-3·70) | 0·33 |
| Teaching hospital 2 | **n=723** | **n=197** |  |  |  |  |
| Tuberculosis | 27/35 (77·1%) | 5/7 (71·4%) | 1·18 (0·33-4·31) | 0·80 | n/a | n/a |
| Cervical cancer or CIN-3 | 4/97 (4·1%) | 5/55 (9·1%) | 1·20 (0·14-10·06) | 0·87 | n/a | n/a |
| Vulvar cancer or VIN-3 | 0/16 (0·0%) | 0/4 (0·0%) | n/a | n/a | n/a | n/a |
| Malignant lymphoma | 99/203 (48·8%) | 40/52 (76·9%) | 1·44 (0·86-2·40) | 0·17 | 1·47 (0·86-2·51) | 0·16 |
| Hepatitis B virus infection | 57/112 (50·9%) | 3/5 (60·0%) | 1·46 (0·41-5·18) | 0·56 | 1·76 (0·48-6·47) | 0·40 |
| Hepatitis C virus infection | 35/47 (74·5%) | 3/6 (50·0%) | 0·68 (0·15-3·00) | 0·61 | n/a | n/a |
| Peripheral neuropathy | 14/213 (6·6%) | 8/68 (11·8%) | 1·34 (0·42-4·28) | 0·62 | n/a | n/a |
| Non-teaching hospital 1 | **n=612** | **n=174** |  |  |  |  |
| Tuberculosis | 26/27 (96·3%) | 10/10 (100·0%) | 1·09 (0·39-3·02) | 0·87 | n/a | n/a |
| Cervical cancer or CIN-3 | 3/158 (1·9%) | 2/62 (3·2%) | 2·15 (0·13-35·42) | 0·59 | n/a | n/a |
| Vulvar cancer or VIN-3 | 0/11 (0·0%) | 0/1 (0·0%) | n/a | n/a | n/a | n/a |
| Malignant lymphoma | 73/113 (64·6%) | 17/20 (85·0%) | 1·13 (0·56-2·28) | 0·73 | n/a | n/a |
| Hepatitis B virus infection | 17/28 (60·7%) | 5/7 (71·4%) | 1·17 (0·33-4·16) | 0·81 | n/a | n/a |
| Hepatitis C virus infection | 5/10 (50·0%) | 0/0 (0·0%) | n/a | n/a | n/a | n/a |
| Peripheral neuropathy | 11/265 (4·2%) | 7/74 (9·5%) | 7·69 (1·30-45·30) | 0·02 | n/a | n/a |

*Analyses are performed using segmented time-series models not correcting for patient characteristics. ** Analyses are performed using multivariable models adjusting for confounding patient characteristics sex, age, socio-economic status, and pregnant at time of indicator condition diagnosis. CIN-3: Cervical intraepithelial neoplasia grade III. n/a: parameter estimates could not be obtained. VIN-3: vulvar intraepithelial neoplasia grade III.

Supplementary table 6: Proportions tested for HIV within 3 months before or after indicator condition diagnosis, and risk ratio, overall and by indicator condition - Random effects model accounting for variability by hospital. Amsterdam region, 2015-2021.

|  | Before intervention (n=6,730) | After intervention (n=1,256) | Adjusted  risk ratio (95% CI)* | p value |
| --- | --- | --- | --- | --- |
| Overall | 2,478/6,730 (36·8%) | 590/1,256 (47·0%) | 1·16 (1·03-1·31) | 0·01 |
| By indicator condition | | | | |
| Tuberculosis | 317/379 (83·6%) | 52/59 (88·1%) | n/a | n/a |
| Cervical cancer or CIN-3 | 46/1,075 (4·3%) | 77/275 (28·0%) | 3·76 (2·01-7·04) | <0·001 |
| Vulvar cancer or VIN-3 | 2/283 (0·7%) | 0/52 (0·0%) | n/a | n/a |
| Malignant lymphoma | 1,021/1,612 (63·3%) | 286/361 (79·2%) | 1·04 (0·88-1·24) | 0·64 |
| Hodgkin's lymphoma | 158/228 (69·3%) | 32/35 (91·4%) | n/a | n/a |
| T-cell lymphoma | 111/173 (64·2%) | 32/36 (88·9%) | n/a | n/a |
| Diffuse large B-cell lymphoma | 393/536 (73·3%) | 132/150 (88·0%) | n/a | n/a |
| Mantle cell lymphoma | 65/90 (72·2%) | 22/26 (84·6%) | n/a | n/a |
| Follicular lymphoma | 121/234 (51·7%) | 18/37 (48·7%) | 0·67 (0·37-1·22) | 0·19 |
| Marginal zone/MALT lymphoma | 58/133 (43·6%) | 21/32 (65·6%) | 1·12 (0·56-2·26) | 0·75 |
| Burkitt lymphoma | 25/28 (89·3%) | 4/4 (100·0%) | n/a | n/a |
| Lymphoplasmacytic lymphoma | 4/16 (25·0%) | 1/1 (100%) | n/a | n/a |
| Non-Hodgkin lymphoma, other | 86/174 (49·4%) | 24/40 (60·0%) | n/a | n/a |
| Hepatitis B virus infection | 520/809 (64·3%) | 77/99 (77·8%) | n/a | n/a |
| Hepatitis C virus infection | 351/499 (70·3%) | 46/63 (73·0%) | n/a | n/a |
| Peripheral neuropathy | 221/2,073 (10·7%) | 52/347 (15·0%) | 2·33 (1·52-3·59) | <0·001 |

*Analyses are performed using multivariable models correcting for relevant patient characteristics sex, age, socio-economic status, and pregnant at time of indicator condition diagnosis. CIN-3: Cervical intraepithelial neoplasia grade III. n/a: parameter estimates could not be obtained. MALT: mucosa-associated lymphoid tissue. VIN-3: vulvar intraepithelial neoplasia grade III.

Supplementary table 7: Characteristics of patients tested and not tested for HIV within 3 months before or after indicator condition diagnosis in five hospitals in the region of Amsterdam, 2015-2021

|  | Tested for HIV  (n=3,068) | Not tested for HIV  (n=4,918) | *p* value |
| --- | --- | --- | --- |
| Sex |  |  | <0·001 |
| Female | 1,285 (41·9%) | 3,203 (65·1%) |  |
| Male | 1,783 (58·1%) | 1,715 (34·9%) |  |
| Pregnant at IC diagnosis* | 98 (7·6%) | 52 (1·6%) | <0·001 |
| Age at IC diagnosis, y | 52 (37-64) | 59 (43-71) | <0·001 |
| Socio-economic status** |  |  | <0·001 |
| Low | 1,204 (39·7%) | 1,693 (34·7%) |  |
| Intermediate | 676 (22·3%) | 1,157 (23·7%) |  |
| High | 1,156 (38·1%) | 2,033 (41·6%) |  |
| Deceased ≤180 days after IC diagnosis | 131 (4·3%) | 113 (2·3%) | <0·001 |

Data are depicted as n (%) or median (IQR). *Percentages are calculated using the number of female patients as denominator. **Overall, 67 patients had a missing socio-economic status value; 32 in the tested and 35 in the not-tested group.

Supplementary table 8: Characteristics of patients testing HIV positive versus HIV negative within 3 months before or after indicator condition diagnosis in five hospitals in the region of Amsterdam, 2015-2021

|  | HIV positive  (n=18) | HIV negative  (n=3,050) | *p* value |
| --- | --- | --- | --- |
| Study period |  |  | 0·23 |
| Pre-intervention | 17 (0·7%) | 2,461 (99·3%) |  |
| Post-intervention | 1 (0·2%) | 589 (99·8%) |  |
| Sex |  |  | 0·10 |
| Female | 4 (0·3%) | 1,281 (99·7%) |  |
| Male | 14 (0·8%) | 1,769 (99·2%) |  |
| Pregnant at IC diagnosis* | 0 (0·0%) | 98 (100%) | 1·00 |
| Age at IC diagnosis, y | 45 (34-54) | 52 (37-64) | 0·05 |
| Socio-economic status** |  |  | 0·42 |
| Low | 10 (0·8%) | 1,194 (99·2%) |  |
| Intermediate | 3 (0·4%) | 673 (99·6%) |  |
| High | 5 (0·4%) | 1,151 (99·6%) |  |
| Indicator condition |  |  | 0·02 |
| Tuberculosis | 8 (2·2%) | 361 (97·8%) |  |
| Cervical cancer or CIN-3 | 0 (0·0%) | 123 (100%) |  |
| Vulvar cancer or VIN-3 | 0 (0·0%) | 2 (100%) |  |
| Malignant lymphoma | 7 (0·5%) | 1,300 (99·5%) | 0·36 |
| Hodgkin's lymphoma | 0 (0·0%) | 190 (100%) |  |
| T-cell lymphoma | 1 (0·7%) | 142 (99·3%) |  |
| Diffuse large B-cell lymphoma | 5 (1·0%) | 520 (99·0%) |  |
| Mantle cell lymphoma | 0 (0·0%) | 87 (100%) |  |
| Follicular lymphoma | 0 (0·0%) | 139 (100%) |  |
| Marginal zone/MALT lymphoma | 0 (0·0%) | 79 (100%) |  |
| Burkitt lymphoma | 1 (3·5%) | 28 (96·6%) |  |
| Lymphoplasmacytic lymphoma | 0 (0·0%) | 5 (100%) |  |
| Non-Hodgkin lymphoma, other | 0 (0·0%) | 110 (100%) |  |
| Hepatitis B virus infection | 2 (0·4%) | 595 (99·7%) |  |
| Hepatitis C virus infection | 1 (0·3%) | 396 (99·8%) |  |
| Peripheral neuropathy | 0 (0·0%) | 273 (100%) |  |
| Hospital of inclusion |  |  | 0·25 |
| University hospital 1 | 4 (0·4%) | 953 (99·6%) |  |
| University hospital 2 | 5 (0·9%) | 585 (99·2%) |  |
| Teaching hospital 1 | 5 (0·5%) | 1,040 (99·5%) |  |
| Teaching hospital 2 | 1 (0·3%) | 299 (99·7%) |  |
| Non-teaching hospital 1 | 3 (1·7%) | 173 (98·3%) |  |
| Died ≤3 months after IC diagnosis | 2 (2·9%) | 68 (97·1%) | 0·01 |
| CD4 count at HIV diagnosis, cells/mm^3^ | 97 (50-130) | n/a |  |
| CD4 count <350 cells/mm^3^ at HIV diagnosis | 17 (94·4%) | n/a |  |

Data are depicted as n (%) or median (IQR). *Percentages are calculated using the number of female patients as denominator. **Overall, 67 patients had a missing socio-economic status value; 32 in the tested and 35 in the not-tested group. CIN-3: Cervical intraepithelial neoplasia grade III. MALT: mucosa-associated lymphoid tissue. n/a: not applicable. VIN-3: vulvar intraepithelial neoplasia grade III.
